# Bypassed and Preserved Stomach Resulted in Superior Glucose Control in Sprague-Dawley Rats with Streptozotocin-Induced Diabetes

**DOI:** 10.1038/s41598-019-46418-y

**Published:** 2019-07-10

**Authors:** Jason Widjaja, Ponnie Robertlee Dolo, Qiang Zhang, Libin Yao, Chao Li, Jian Hong, Hui Wang, Song Meng, Yong Shao, Xiaocheng Zhu

**Affiliations:** 1 Department of General Surgery, the Affiliated Hospital of Xuzhou Medical University, Xuzhou, Jiangsu 221002 P.R. China; 2 Institute of Digestive Diseases, Xuzhou Medical University, Xuzhou, Jiangsu 221002 P.R. China

**Keywords:** Metabolic syndrome, Stomach

## Abstract

Recent studies suggest the possibility of the stomach playing a role in diabetes remission after bariatric surgery. In this study, we investigated whether bypassing the stomach alleviates diabetes in diabetic rodent model. Eighteen moderately obese and diabetic Sprague-Dawley rats were randomly assigned to Esophagoduodenostomy with or without gastric preservation (EDG and EDNG/total gastrectomy, respectively), and SHAM groups. Bodyweight, food intake, fasting glucose level, oral glucose tolerance test result (OGTT), and hormone levels (insulin, glucagon-like peptide-1, ghrelin, gastrin and glucagon) were measured preoperative and postoperatively. Postoperatively, bodyweight and food intake did not differ significantly between the EDG and EDNG groups. Postoperative fasting blood glucose and OGTT results declined significantly in the EDG and EDNG group when compared with the respective preoperative levels. Postoperative glucose control improvements in EDNG group was significantly inferior when compared to EDG. Compared preoperatively, postoperative plasma ghrelin and gastrin levels declined significantly in EDNG group. Preoperative and postoperative plasma GLP-1 level did not differ significantly among all the groups. Postoperatively, EDG group had significantly higher insulin and lower glucagon levels when compared with SHAM. In conclusion, bypassing and preserving the stomach resulted in superior glucose control improvements than total gastrectomy.

## Introduction

Obesity and obesity-related diabetes have become major problems worldwide. A study in 2004 by Wild S *et al*. estimated that the global population with diabetes was around 171 million in 2000 and that it will continue to increase and reach 366 million by 2030^1^. Bariatric surgery has become an important method in treating obesity and diabetes, and was reported to have superior efficacy than drug therapy^2^. In bariatric surgery, roux-en-y gastric bypass (RYGB) and sleeve gastrectomy (SG) have become the most popular procedures performed, and are effective in inducing diabetes remission^3^.

The anti-diabetic effect of RYGB can be elucidated through foregut (proximal gut exclusion) and hindgut (rapid nutrients flow to the distal gut) theories^4^. Hindgut theory holds that anti-diabetic effect of bariatric surgery was achieved by expediting delivery of nutrient to the distal intestine, enhancing incretin hormone level (glucagon-like peptide 1, GLP-1, has been proposed as the most potent candidate)^4^. Alternatively, foregut theory proposed that the anti-diabetic effect of bariatric surgery depends on exclusion of the duodenum and proximal jejunum from the transit of nutrients, possibly to decrease the anti-incretin hormone level^4^.

However, the diabetes remission outcomes following a stand-alone foregut exclusion procedure, such as duodenal-jejunal bypass (DJB), remains inconclusive. While some studies have reported that DJB resulted in effective anti-diabetic outcomes^5,6^, several other studies have also suggested that DJB is still ineffective to induce diabetes remission^7–10^. The unconvincing reports on the anti-diabetic effect of DJB is interesting because RYGB continued to demonstrate significant diabetes remission outcomes^11^. It is important to note that RYGB procedure resulted in bypassing not only the proximal gut, but also the majority of the stomach. Accordingly, we presumed that the stomach might play a key role in glucose homeostasis.

Indeed, several studies have suggested that the stomach might play a key role in glucose homeostasis, though remains inconclusive. Some studies reported that total gastrectomy resulted in impaired glucose control^12–16^, whereas others reported that total gastrectomy resulted in diabetes remission in gastric cancer and type-2 diabetes mellitus (T2DM) patients^17–19^.

**Figure 1:**
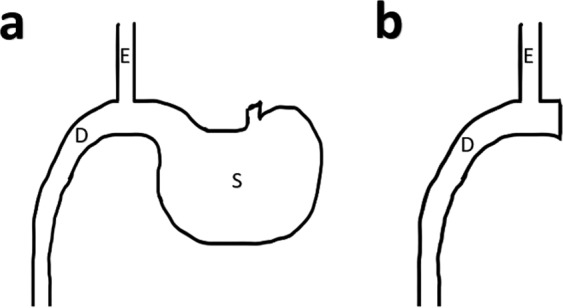
Illustrates (**a**). Esophagoduodenostomy with gastric preservation (EDG), (**b**) Esophagoduodenostomy without gastric preservation (EDNG). E = esophagus; D = duodenum; S = stomach.

This experiment aimed to further investigate the potential role of the stomach on glucose control. In this study, using T2DM-induced Sprague-Dawley (SD) rodent model, we investigate the anti-diabetic effect of bypassing the stomach (without foregut exclusion), with either preserved gastric (esophagoduodenostomy with gastric preservation, EDG) or total gastrectomy (esophagoduodenostomy without gastric preservation, EDNG) (Fig. 1).

## Results

### Bodyweight and food intake

**Figure 2:**
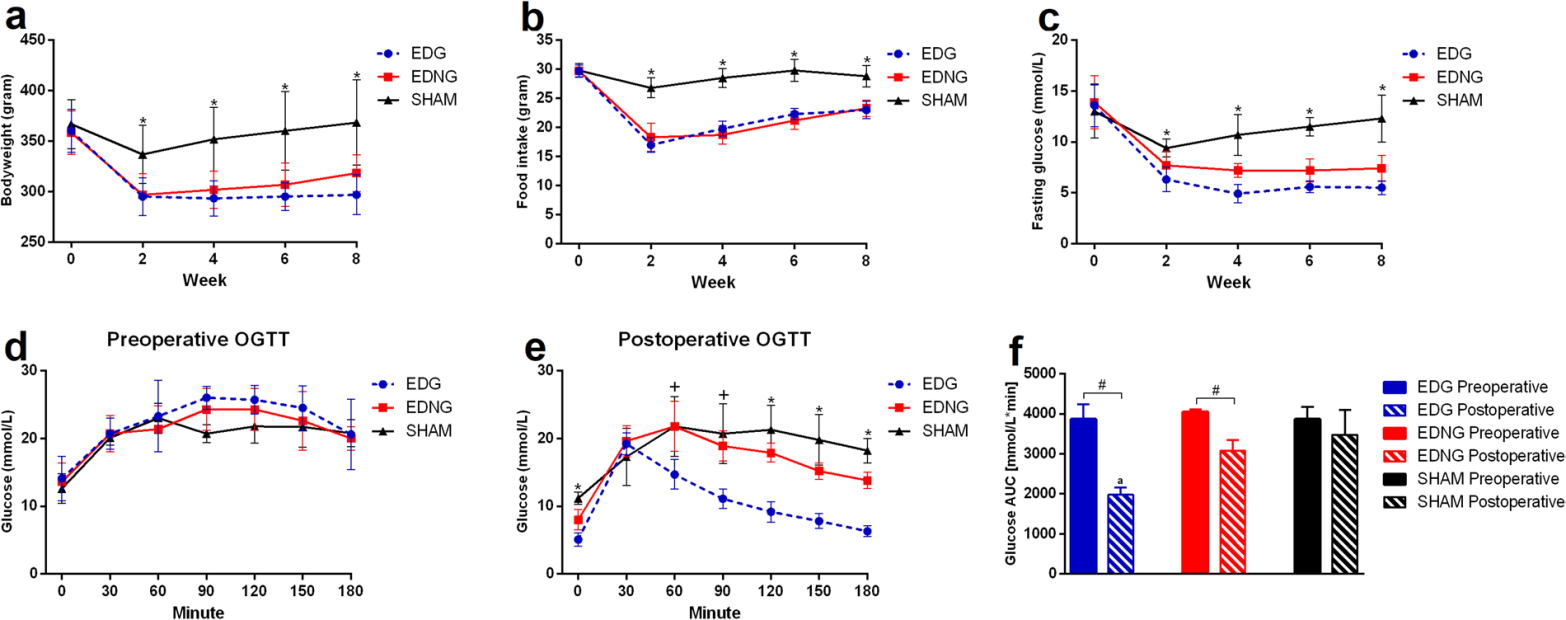
Illustrates changes in body weight (**a**), food intake (**b**), fasting glucose level (**c**) among all the groups. Oral glucose tolerance test (OGTT) results preoperative (**d**) and postoperatively (**e**), and area under the curve (AUC) for glucose level (**f**) are presented. *Significant EDG and EDNG compared with SHAM (*p*-value < 0.05). ^+^Significant EDG compared with SHAM (*p*-value < 0.05). ^#^Significant preoperative compared with postoperative value (*p*-value < 0.05). ^a^Significant postoperative EDG compared with postoperative EDNG and SHAM (*p*-value < 0.05).

There were no significant differences in bodyweight and food intake among all groups preoperatively. Throughout postoperative period, EDG and EDNG showed significantly lower bodyweight and food intake compared to SHAM (p-value < 0.05) (Fig. 2a,b). At postoperative 8-week, there were no statistical differences between EDG and EDNG in mean bodyweight loss (17 ± 4 and 11 ± 4% respectively, from preoperative bodyweight) and food intake reduction (22 ± 5 and 21 ± 5% respectively, from preoperative food intake).

### Glucose level (Fasting blood glucose and oral glucose tolerance test)

**Figure 3:**
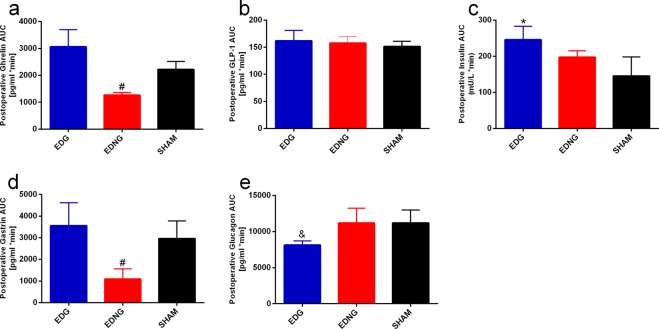
Illustrates postoperatively, the hormone level assessment of plasma ghrelin (**a**), GLP-1 (**b**), insulin (**c**), gastrin (**d**) and glucagon (**e**). ^#^Significant EDNG compared with EDG and SHAM (*p*-value < 0.05). *Significant EDG compared with SHAM (*p*-value < 0.05). ^&^Significant EDG compared with EDNG and SHAM (*p*-value < 0.05).

There were no significant differences in fasting blood glucose (FBG) and oral glucose tolerance test (OGTT) result among all groups preoperatively. Throughout postoperative period, FBG level declined significantly in EDG and EDNG group when compared with the respective preoperative level (p-value < 0.05) (Fig. 2c). At postoperative 8-week, FBG levels declined significantly from preoperative level by 58 ± 10% and 45 ± 15% in EDG and EDNG group respectively (p-value < 0.05). Compared preoperatively, postoperative EDG and EDNG showed significant improvements in glucose control (p-value < 0.05) (Fig. 3f). Postoperatively, EDG showed significantly superior glucose control when compared with EDNG and SHAM (p-value < 0.05) (Fig. 3f). SHAM group preoperative and postoperative results did not differ significantly.

### Ghrelin

There were no significant differences in ghrelin level among all groups preoperatively (Table 1). Compared preoperatively, postoperative EDNG group ghrelin level declined significantly (p-value < 0.05). Preoperative and postoperative ghrelin level did not differ significantly in the EDG and SHAM group. Postoperative ghrelin AUC level was significantly lower in EDNG group when compared with EDG and SHAM (Fig. 3a).

**Table 1:** Hormone levels among groups preoperative and postoperatively. Data are shown as mean ± S.E.M.

Preoperative and Postoperative Hormones Level						
	Preoperative			Postoperative		
**Ghrelin (pg/ml)**						
	0 min	30 min	60 min	0 min	30 min	60 min
EDG	47.90 ± 3.2	42.3 ± 7.0	44.8 ± 5.5	42.5 ± 12.7	50.3 ± 13.5	51.7 ± 3.5
EDNG	43.8 ± 12.0	36.9 ± 6.5	41.4 ± 15.2	19.49 ± 3.5*	16.2 ± 5.7*	19.7 ± 6.3*
SHAM	38.3 ± 9.4	33.7 ± 10.5	32.7 ± 9.7	37.3 ± 5.1	43.3 ± 16.2	33.1 ± 8.6
**GLP-1 (pg/ml)**						
	0 min	30 min	60 min	0 min	30 min	60 min
EDG	2.1 ± 0.24	2.3 ± 0.52	2.4 ± 0.53	2.3 ± 0.36	2.7 ± 0.17	2.5 ± 0.54
EDNG	2.0 ± 0.10	2.3 ± 0.37	2.3 ± 0.42	2.1 ± 0.16	2.7 ± 0.49	2.2 ± 0.46
SHAM	1.9 ± 0.54	2.2 ± 0.90	2.2 ± 0.34	2.4 ± 0.17	2.6 ± 0.31	2.6 ± 0.24
**Insulin (mU/L)**						
	0 min	30 min	60 min	0 min	30 min	60 min
EDG	5.9 ± 1.00	5.7 ± 0.71	6.2 ± 0.22	4.2 ± 0.80*	4.2 ± 0.95	3.9 ± 0.42*
EDNG	5.9 ± 0.57	5.4 ± 0.32	4.5 ± 0.42	3.3 ± 0.50*	3.3 ± 0.41*	3.2 ± 0.37*
SHAM	5.5 ± 0.30	4.9 ± 0.45	4.2 ± 1.66	2.9 ± 0.38*	2.3 ± 1.3*	2.3 ± 1.36
**Gastrin (pg/ml)**						
	0 min	30 min	60 min	0 min	30 min	60 min
EDG	53.6 ± 14.6	75.0 ± 4.4	53.2 ± 9.9	62.9 ± 15.1	57.3 ± 19.7	52.1 ± 16.3
EDNG	49.8 ± 9.5	64.4 ± 21.3	49.3 ± 5.8	27.1 ± 9.9*	15.4 ± 9.0*	16.2 ± 5.0*
SHAM	57.2 ± 10.2	53.9 ± 18.2	49.2 ± 7.9	55.0 ± 7.7	49.8 ± 14.1	43.4 ± 13.1
**Glucagon (pg/ml)**						
	0 min	30 min	60 min	0 min	30 min	60 min
EDG	188.8 ± 29.0	157.2 ± 25.3	173.2 ± 45.4	127.3 ± 9.7*	146.5 ± 22.0	121.6 ± 13.7
EDNG	170.2 ± 19.0	173.5 ± 34.4	197.7 ± 15.0	191.7 ± 32.8	192.8 ± 59.8	169.0 ± 42.6
SHAM	170.9 ± 16.6	141.9 ± 32.4	170.7 ± 36.1	186.7 ± 28.7	165.3 ± 42.0	187.2 ± 20.9

*Significant difference compared with the respective preoperative level at that particular interval (*p*-value < 0.05).

### GLP-1

Compared preoperatively, EDG and EDNG showed slight elevation in GLP-1 level postoperatively, but without statistical significance (Table 1). Preoperative and postoperative GLP-1 level did not differ significantly in the SHAM group. Postoperative GLP-1 AUC level did not show significant difference among all the groups (Fig. 3b).

### Insulin

Preoperative and postoperative insulin level differs significantly among all the groups (p-value < 0.05) (Table 1). Postoperatively, EDG group insulin level was significantly higher when compared with SHAM group (Fig. 3c) (p-value < 0.05). Postoperative insulin level did not differ significantly between EDNG and SHAM groups.

### Gastrin

There were no significant differences in gastrin level among all groups preoperatively (Table 1). Compared preoperatively, postoperative EDNG group gastrin level declined significantly (p-value < 0.05). Preoperative and postoperative gastrin level did not differ significantly in the EDG and SHAM group. Postoperative gastrin AUC level was significantly lower in EDNG group when compared with EDG and SHAM (Fig. 3d).

### Glucagon

There were no significant differences in glucagon level among all groups preoperatively (Table 1). Compared preoperatively, postoperative fasting glucagon level declined significantly in EDG group (p-value < 0.05). Preoperative and postoperative glucagon level did not differ significantly in the EDNG and SHAM group. Postoperative glucagon AUC level was significantly lower in EDG group when compared with EDNG and SHAM (Fig. 3e).

## Discussion

Our particular study showed that bypassing the stomach alone resulted in significant improvements on glucose control. Furthermore, total gastrectomy (EDNG) resulted in significantly inferior glucose control improvements compared with bypassed and preserved stomach (EDG).

Both EDG and EDNG postoperative FBG and glucose AUC level declined significantly compared to the respective preoperative level (Fig. 2). But interestingly postoperative EDNG group had significantly inferior glucose control compared to EDG. These findings could have two implications. First, bypassing the stomach alone (with either preserved stomach or total gastrectomy) able to significantly improved glucose control. Subsequently, it is essential to have a bypassed and preserved stomach for superior anti-diabetic effect.

Several studies have also reported that total gastrectomy could resulted in impaired glucose control^12–16^. Animal studies have reported that total gastrectomy resulted in impaired glucose tolerance, possibly due to delayed insulin and increased glucagon release^12,13^. Clinically, whilst some studies reported that total gastrectomy resulted in diabetes remission^17–19^, others have reported unconvincing results. Friess H *et al*. reported that post-gastrectomized patients had a pathological glucose tolerance with increased postprandial insulin and glucagon secretion^14^. Yamamoto H *et al*. reported high incidence of post-intervention hyperglycemia in the patients after a total gastrectomy^15^. Ito K *et al*. also suggested that gastrectomy might lead to increase in glucagon and glucose absorption level resulting in hyperglycemia^20^. Finally, Hayashi SY *et al*. reported a high rate of refractory cases in diabetic and gastric cancer patients, and also high rate of new-onset diabetes cases in non-diabetic and gastric cancer patients, following roux-en-y gastrectomy^21^. Our study similarly demonstrated that EDNG (total gastrectomy) resulted in lower insulin, higher glucagon and subsequently inferior glucose control level when compared to EDG (bypassed and preserved stomach).

The hormone ghrelin is prominently secreted in the stomach, functioning to regulate food intake and energy homeostasis^22,23^. Our study demonstrated that even with higher level of ghrelin in EDG group, compared to EDNG group, food intake was reduced and glucose control was also achieved. Although it is understood that lower ghrelin level is associated with lower food intake and thus weight loss^24,25^, other hormonal alterations (GLP-1, obestatin) might have also affected the signalling of ‘hunger’, leading to a reduction in food intake^26^. Several studies have also reported possible association between ghrelin and glucose control. Ruama J *et al*. reported that ghrelin cell density was reduced in pre-diabetic and diabetic conditions^27^. Moreover, ghrelin might promote GLP-1 secretion in response to meals^28^ and might have protective effects on the pancreas^29^. Overexpression of ghrelin following DJB was found to be associated with diabetes alleviation^30^. Nonetheless, our current study did not find correlation between ghrelin level and glucose control.

Improved glucose control after bariatric surgery is often correlated with elevated GLP-1 level^31–33^. Our experiment failed to find significant elevation of GLP-1 in all the groups postoperatively, even though the EDG group demonstrate significantly superior glucose control improvements, suggesting that GLP-1 might not be a key factor for glucose control improvements in T2DM subjects. Indeed, some studies have proposed that the hormone GLP-1 might not be the most important factor for diabetes remission following bariatric surgery^34,35^.

Studies in both animal and human have reported that GLP-1 receptor (GLP-1r) is expressed in the stomach^36,37^, and was found to be reduced in T2DM^38^. Whether GLP-1r in the stomach is closely related with glucose homeostasis is still unknown, but some evidence exists. GLP-1 intervention on an isolated perfused rat stomach have shown to enhanced the release of somatostatin^39–41^, a known regulator of insulin and glucagon^42^. Studies have suggested that somatostatin might be able to improved glucose control, possibly by suppressing glucagon secretion^43–45^. Nonetheless, the role of GLP-1r in the stomach on glucose homeostasis is still far from being clear. Whether reduced GLP-1r in the stomach is a result of diabetes? Or related with the development of diabetes? These questions should be addressed in the future.

The hormone gastrin has been suggested to have an incretin-like stimulating actions and thus might improve glucose control in diabetic subjects^46^. The use of proton-pump inhibitors (PPI), that subsequently resulted in hypergastrinemia, was reported to improved glucose control in diabetic patients^47^. According to our study, we did not find evidence that hypergastrinemia might improve glucose control, as SHAM and EDG groups had similar level of plasma gastrin postoperatively, and yet only EDG group had glucose control improvements. Hypergastrinemia was also not found clinically following RYGB and SG procedures^48,49^, though one animal study suggested otherwise^50^. Furthermore, the effect of PPI on diabetes is still controversial, as one study did not found improvements on the glycated haemoglobin following interventions^51^. Further studies on the use of PPI on diabetic patients will be needed, especially considering different variables such as the “degree” of the diabetes and the subsequent B-cell function.

Our study was not without limitations. The number of rodents used was not high (n = 6, for each group). Furthermore, we only observed the postoperative outcomes of 8 weeks.

We conclude that bypassing the stomach alone resulted in significant improvement on glucose control. Subsequently, following stomach bypassed, the removal of the gastric remnant (total gastrectomy) resulted in significantly inferior glucose control improvement, when compared with the bypassed and preserved stomach. We believed that our study complements the anatomical understanding in that the stomach seemed to have a key role in glucose homeostasis. Further studies will be needed in order to improve our understanding on the physiological aspects.

## Materials and Methods

### Animals

This study was approved by the ethics committee of Xuzhou Medical University Research Animal Centre. All applicable institutional and national guidelines of the People’s Republic of China for the care and use of animals were followed.

8–10 weeks old male Sprague-Dawley (SD) rats were purchased from the Xuzhou Medical University Research Animal Centre. Free access to water and dry chow was provided. Constant temperature and humidity with 12 hours day/12 hours night cycle were maintained throughout the study. Type 2 diabetes model was induced through high-fat diet and intraperitoneal injection of low-dose streptozotocin (STZ, 35 mg/kg). Random blood glucose levels were measured 72 hours following STZ injection with a hand-held glucose meter. Rats with random blood glucose level > 16.0 mmol/L in 3 consecutive days were considered to be diabetic.

### Study design

18 diabetic male SD rats were randomly assigned into 3 different groups: 1) Esophagoduodenostomy with gastric preservation (EDG, n = 6), 2) Esophagoduodenostomy without gastric preservation (EDNG, n = 6), and 3) SHAM (n = 6) (Fig. 1). Bodyweight, food intake, and fasting blood glucose (FBG) levels were measured preoperatively and postoperatively at the 2^nd^, 4^th^, 6^th^ and 8^th^ week. Oral glucose tolerance test (OGTT) was performed preoperatively and at postoperative 8^th^ week. Blood samples were taken preoperatively and at postoperative 8^th^ week for hormonal analysis (Ghrelin, Insulin, GLP-1, Gastrin and Glucagon). Glucose measurements and blood sampling was performed at 9 a.m., approximately 12 hours following fasting.

### OGTT and blood samples collection

2-hour OGTT with gavage of 50% glucose solution (3 mg/kg) were performed preoperatively and at postoperative 8^th^ week in all groups. Blood glucose was measured from the tail vein at 0, 30, 60, 90, 120, 150 and 180 min using a hand-held glucometer (approximately 1 µl of blood samples were obtained per sampling) in conscious rats.

During retroorbital blood sampling, rats were anaesthetized briefly with inhaled anaesthetic agent isoflurane using “drop jar” method with cotton as the absorbent^52^. The use of isoflurane resulted in the rats being anaesthetized for approximately 90–120 seconds, sufficient for a brief blood sampling procedure. 20 µl sized microhematocrit tubes were used to obtained the blood samples to minimize the risk of injury. Approximately 0.5 ml of retroorbital blood samples were taken at before (0 min) then 30 and 60 min following gavage (Ensure 7.68 ml/kg) in unconscious rats, followed by centrifugation (3000 rpm for 10 minutes) to collect the plasma and stored at −80 degree Celsius until further use. Hormonal analysis was performed using enzyme-linked immunosorbent assay kit (ELISA kit, Shanghai Jianglai industrial Limited By Share Ltd).

### Surgical procedure

All groups: Overnight fasting was ensured before surgery. Following anaesthesia (5% chloral hydrate 0.5 mL/100 g, intraperitoneal), each rat was placed on the operating table and the abdomen was cleaned using 5% povidone-iodine. Midline incision was approximately 3 cm in length. Abdominal closure was performed using 2–0 Mersilk suture with a continuous suture technique.

EDG and EDNG groups received identical esophagoduodenostomy, with the EDG group having complete gastric preservation whereas the EDNG group underwent total gastrectomy (Fig. 1). The duodenum was located approximately 5 mm distal to the pylorus, esophagoduodenostomy was then performed as an end-to-side anastomosis using 6–0 silk suture with continuous suturing technique (Ningbo Medical Needle Co., Ltd., Ningbo, China).

SHAM rat esophagus was located, transected and then re-anastomosed using 6–0 silk suture with continuous suturing technique (Ningbo Medical Needle Co., Ltd., Ningbo, China) to mimic similar traumatic manipulation.

All rats were allowed free access to water 24-hour following surgery, followed by free access to normal chow 48-hour following surgery.

### Statistical analysis

All data are presented as mean ± SD; area under the curve (AUC) was calculated using the trapezoidal method (Graphpad Prism 6). A student t test was used to compare differences between mean. One-way ANOVA analysis was used to compare surgical groups. All tests were two tailed and considered statistically significant with p < 0.05.

## Data Availability

The datasets generated during and/or analysed during the current study are available from the corresponding author on request.
